# A Straightforward Interpretation of Proximity Labeling through Direct Biotinylation Analysis

**DOI:** 10.1021/acsomega.5c03099

**Published:** 2025-06-11

**Authors:** Han Byeol Kim, Kwang-eun Kim

**Affiliations:** † Organelle Medicine Research Center, 37974 Yonsei University Wonju College of Medicine, Wonju 26426, Republic of Korea; ‡ Department of Convergence Medicine, 37974 Yonsei University Wonju College of Medicine, Wonju 26426, Republic of Korea; § Department of Global Medical Science, 37974 Yonsei University Wonju College of Medicine, Wonju 26426, Republic of Korea

## Abstract

Proximity labeling (PL) is a revolutionary tool in proteomics, enabling the precise identification of protein interactions in live cells. However, conventional statistical approaches for analyzing biotinylation data often lead to false positives, hindering the accuracy of the PL studies. In this study, we propose a direct biotinylation analysis approach that focuses on identifying only biotinylated peptides rather than relying solely on statistical comparisons. Using LC-MS data from a prior TurboID-based study, we reanalyzed secretome data sets and demonstrated significant improvements in identifying true biotinylated proteins with fewer false positives. By applying this approach to tissue-specific secretome data, we identified fibronectin (FN1) as a pericyte-specific marker. Our findings highlight that the limitations of traditional methods are insufficiently robust, and we advocate for the adoption of direct biotinylation analysis to enhance data reliability in PL-based proteomics. This methodology sets a new standard for studying protein interactions and secretomes, offering deeper insights into cellular- and tissue-specific molecular networks.

## Graphical abstract

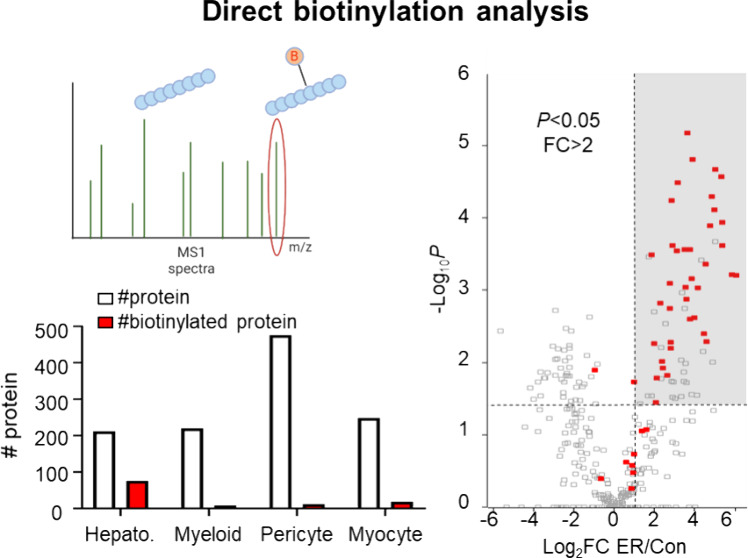

## Introduction

Proximity labeling is a versatile technique that employs enzymes capable of modifying specific small molecules on proteins to label neighboring proteins located close to a target protein.^1,2^ This method has proven valuable for studying protein interaction networks and various other biological processes.^3–6^ In recent years, TurboID, a biotin ligase engineered for high enzymatic efficiency, has gained popularity in proximity labeling applications.^7–11^ While traditionally employed to identify binding partners of specific proteins, TurboID-based proximity labeling has expanded to studies that investigate cellular localization by enabling the selective labeling of specific cells or organelles within cells. One notable application of this expanded use is the construction of engineered in vivo systems in which TurboID is specifically expressed in the endoplasmic reticulum (ER) of targeted tissues and cell types. This enables tissue- and cell-specific biotinylation of ER-resident proteins, allowing researchers to extract and identify biotin-labeled secreted proteins from blood samples to study cell-specific secretomes.^12–18^ Traditional in vivo secretome studies have faced significant limitations in identifying the origins of secreted proteins within specific tissues.^19,20^ The development of TurboID-based proximity labeling holds promise for overcoming these challenges, offering a new frontier in secretome research by enabling more precise tracking of secreted proteins to their tissue and cellular origins.

Conventional studies on enriching and analyzing biotinylated proteins have typically used statistical approaches to statistically confirm quantitative increases in enriched proteins, thereby identifying biotinylated proteins. However, these methods often detect both biotinylated and nonbiotinylated peptides when identifying biotin-labeled proteins, which introduces the potential for various false positives. The primary contributors to these false positives include nonbiotinylated peptides that originate from binding proteins that form complexes with biotin-labeled proteins or proteins that nonspecifically bind to enrichment beads. To minimize the probability of false positive identifications in biotin-labeled proteins, recent advances have introduced methods that specifically target biotinylated peptides during enrichment or systematically identify biotinylation sites using advanced liquid chromatography–mass spectrometry (LC–MS) techniques, such as Spot-ID and SR-PL.^21–23^

In TurboID-based proximity labeling for biotinylation proteomics analysis, TurboID is fused to the bait protein for promoting proximity-based biotinylation of nearby proteins within a defined interaction radius (Figure 1). Upon biotin treatment, the introduced TurboID initiates the covalent attachment of biotin molecules to lysine residues on proteins in close proximity to TurboID. Following biotinylation, the labeled proteins are enriched through affinity capture using beads conjugated with streptavidin or similar proteins that exhibit strong binding affinity to biotin. This enrichment step isolates the biotinylated proteins from the complex cellular mixture, allowing for a more focused proteomic analysis. To prepare the enriched proteins for liquid chromatography–mass spectrometry (LC–MS)-based proteomic analysis, an on-bead digestion is typically employed. In this process, a digestion enzyme, such as trypsin, is directly applied to the biotinylated proteins bound to the streptavidin-conjugated beads, cleaving the proteins into peptides while biotinylated peptides remain attached to the beads. Subsequent to on-bead digestion, the cleaved peptides can be collected and processed for LC–MS analysis to identify and quantify the biotinylated proteins. Alternatively, an additional elution step can be applied to release the biotinylated peptides from the beads and they are pooled with the on-bead digested peptides, resulting in a comprehensive mixture for LC–MS analysis^12,13,24^ (Figure 1).

**Figure 1:**
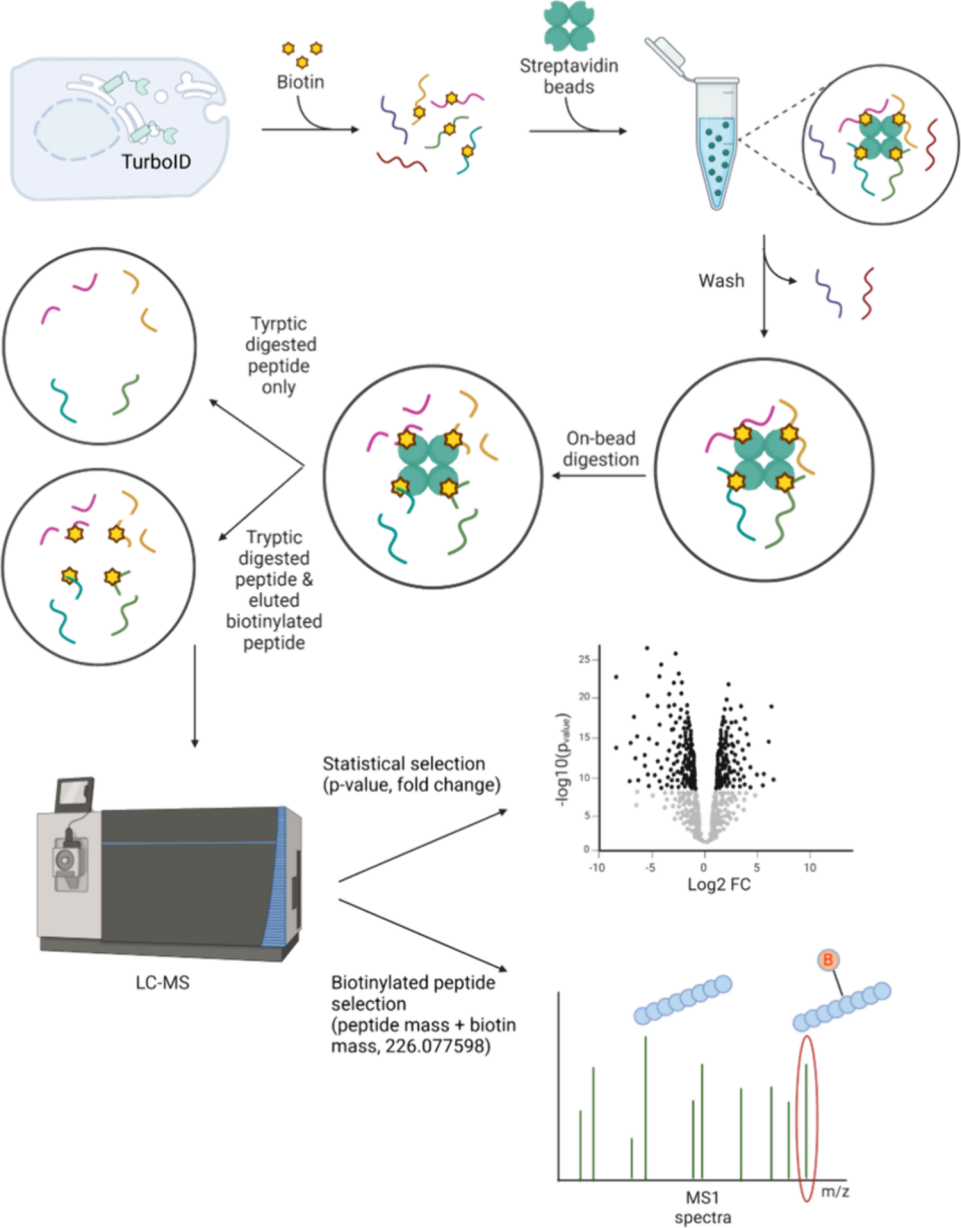
Analysis workflow for direct analysis of biotinylation.

Theoretical workflow illustrating the identification of TurboID-based biotin-labeled proteins using LC-MS. Sequential process includes peptide enrichment from biotin-labeled proteins, followed by identification through conventional statistical approaches and direct analysis. Created with BioRender.com.

However, when samples prepared through these conventional methods are analyzed by LC–MS, both biotinylated and nonbiotinylated peptides can be detected. To distinguish and exclude these false nonbiotinylated peptides from true biotinylated proteins, conventional studies commonly employ statistical approaches with negative controls. Nonetheless, as shown in the study by Shin et al.,^22^ these approaches have demonstrated limitations in successfully excluding nonbiotinylated. Even a stringent washing step with 2 M urea, as employed in their study, was not sufficient to completely eliminate these nonbiotinylated peptides. To minimize such false positive results, analytical approaches such as SR-PL and BioSITe, which enrich biotinylated peptides at the peptide level and allow selective analysis of truly biotinylated peptides, have begun to be adopted in various studies.^12,25–28^ Building on this approach, we propose that false positive results can be effectively minimized by focusing on only biotinylated peptides during the LC-MS data search in conventionally prepared samples.

In this study, we explored the potential of applying this peptide-specific LC-MS data search approach to enhance the accuracy of the results. To implement this approach, we utilized dataset PXD021602 from the study by Wei et al.^13^, which investigated the cell type-selective secretome. Their dataset was useful for comparison because they captured biotinylated proteins using streptavidin beads but did not carry out trypsin digestion on the beads. Instead, they eluted the bound proteins by boiling and subsequently transferred them to S-Trap micro columns, where trypsin digestion was conducted. We proposed our refined method as a direct biotinylation secretome analysis.

## Results

### Reanalysis of the Hepatocyte Subcellular Secretome

Wei et al.^13^ developed an in vivo labeling system for the hepatocyte secretome by designing AAV (adeno-associated virus) constructs driven by the hepatocyte-specific Tbg promoter. These constructs expressed TurboID variants targeted to the cytosol, ER, or membrane through the introduction of NES, KDEL, or PDGFRb-TM localization sequences (Figure 2a). They introduced the resulting AAVs into mice via tail vein injection to induce the expression of each TurboID variant specifically in the liver, followed by biotin administration to initiate biotin labeling mediated by TurboID (Figure 2b). Blood samples collected from these mice were subjected to biotinylated protein enrichment, and the enriched samples were analyzed by LC-MS.

**Figure 2:**
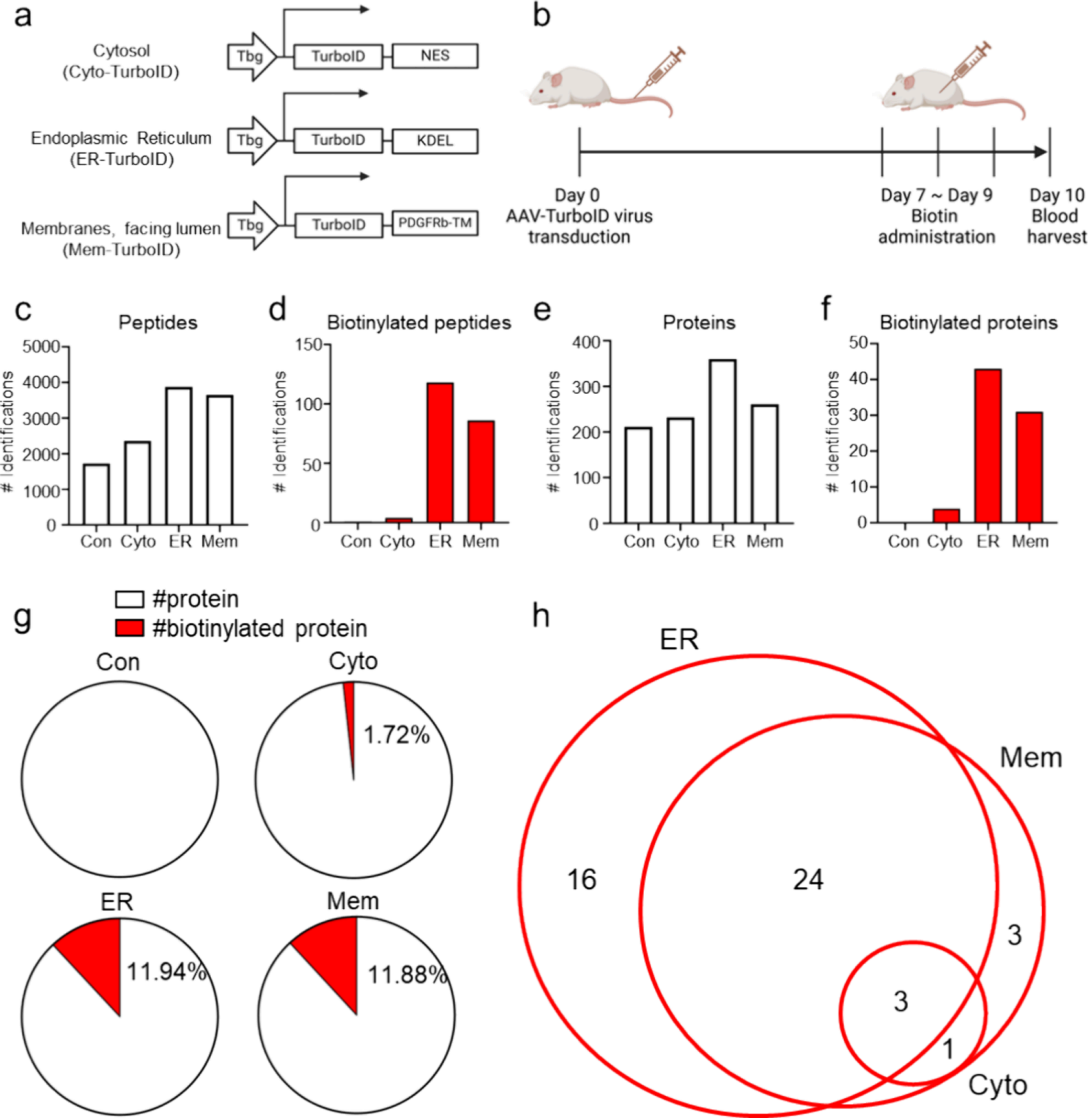
Reanalysis of hepatocyte subcellular secretome. (a) Schematic of the AAV construct expressing TurboID specifically in hepatocytes. (b) Timeline schematic illustrating the in vivo labeling system setup for the hepatocyte-specific secretome, with vertical lines indicating the time points of experimental procedures. (c) Number of total peptides identified in each in vivo system through reanalysis. (d) Number of biotinylated peptides. (e) Number of total proteins. (f) Number of biotinylated proteins. Con, Cyto, ER, and Mem represent data from the control, cyto-TurboID, ER-TurboID, and Mem-TurboID introduced in vivo labeling systems, respectively. (g) Pie chart showing the proportion of biotinylated proteins among all identified proteins. (h) Venn diagram of biotinylated proteins identified across each in vivo labeling system. Created with BioRender.com.

Using the raw LC-MS data files (PXD021602) generated from these experiments, we conducted a proteomics data reanalysis. A key difference between our approach and that of Wei et al.^13^ was the inclusion of a dynamic modification parameter for biotinylation (+226.078 Da) on lysine side chains to directly identify biotinylated peptides. We organized the reanalysis results at both the protein and peptide tables (Tables S1 and S2).

In the direct biotinylation secretome analysis, the biotinylated peptide to unbiotinylated peptide ratio was minimal in samples derived from control mice (without TurboID construct) and cyto-TurboID mice, showing ratios of 1/1722 and 4/2356, respectively. In contrast, ER-TurboID and Mem-TurboID samples exhibited relatively higher biotinylated to unbiotinylated peptide ratios of 118/3882 and 86/3658, respectively (Figure 2c,d, and Tabled S2). At the protein level, similar trends were observed, with control, cyto-TurboID, ER-TurboID, and Mem-TurboID samples showing biotinylated to unbiotinylated protein ratios of 0/211, 4/232, 43/360, and 31/261, respectively (Figure 2e,f and Table S1). These findings aligned with our expectations, as ER-TurboID and Mem-TurboID were anticipated to label secreted proteins selectively. However, despite the enrichment of biotinylated proteins facilitated by ER-TurboID and Mem-TurboID constructs, only 11.94% and 11.88% of all proteins identified by mass spectrometry in these samples were biotinylated, respectively (Figure 2g). In addition, all proteins identified in the cyto-TurboID samples were also detected in the ER-TurboID and Mem-TurboID samples, with no proteins uniquely identified in the cyto-TurboID samples (Figure 2h). This result suggested that a substantial proportion of unbiotinylated proteins could still be identified even after enrichment. We assume that these unbiotinylated proteins could contribute to false-positive results, potentially impacting the accuracy of the study findings.

### Direct Analysis of Biotinylated Secretome

As we mentioned previously, conventional analysis methods aim to exclude false positives and identify true positives by preparing sufficient biological replicates and employing statistical approaches that leverage fold change and *p*-value comparisons with negative controls for statistical analysis. First, we reanalyzed the searched proteomics data based on these conventional statistical methods, comparing negative controls with ER-TurboID results to generate fold changes and *p*-values, and visualized the data in volcano plots (Figure 3a–c). Various criteria were applied to these results, including a low cutoff (fold change > 0), a standard cutoff (fold change >2, *p*-value < 0.05), and a high cutoff (fold change > 8, *p*-value < 0.005), to assess the potential true positive outcomes under conventional approaches. Additionally, we highlighted proteins identified as directly biotinylated in the direct biotinylation secretome analysis with red dots (Figure 3a–c). Using the low, standard, and high cutoffs, we observed 184, 66, and 27 proteins identified, respectively (Figure 3d). In contrast, the number of proteins identified as directly biotinylated proteins at each cutoff was 46, 38, and 23, respectively (Figure 3d). Notably, under the standard cutoff commonly used in statistical approaches, 28 of the 66 proteins identified were classified as false peptides by direct biotinylation secretome analysis (Figure 3d). While applying the stricter high cutoff criterion reduced the number of false-positive proteins, it also significantly reduced the number of proteins identified. Moreover, we found that 4.2, 20.8, and 52.1% of all directly biotinylated proteins were unintentionally excluded under the low, standard, and high cutoffs of the traditional statistical approach, respectively (Figure 3e).

**Figure 3:**
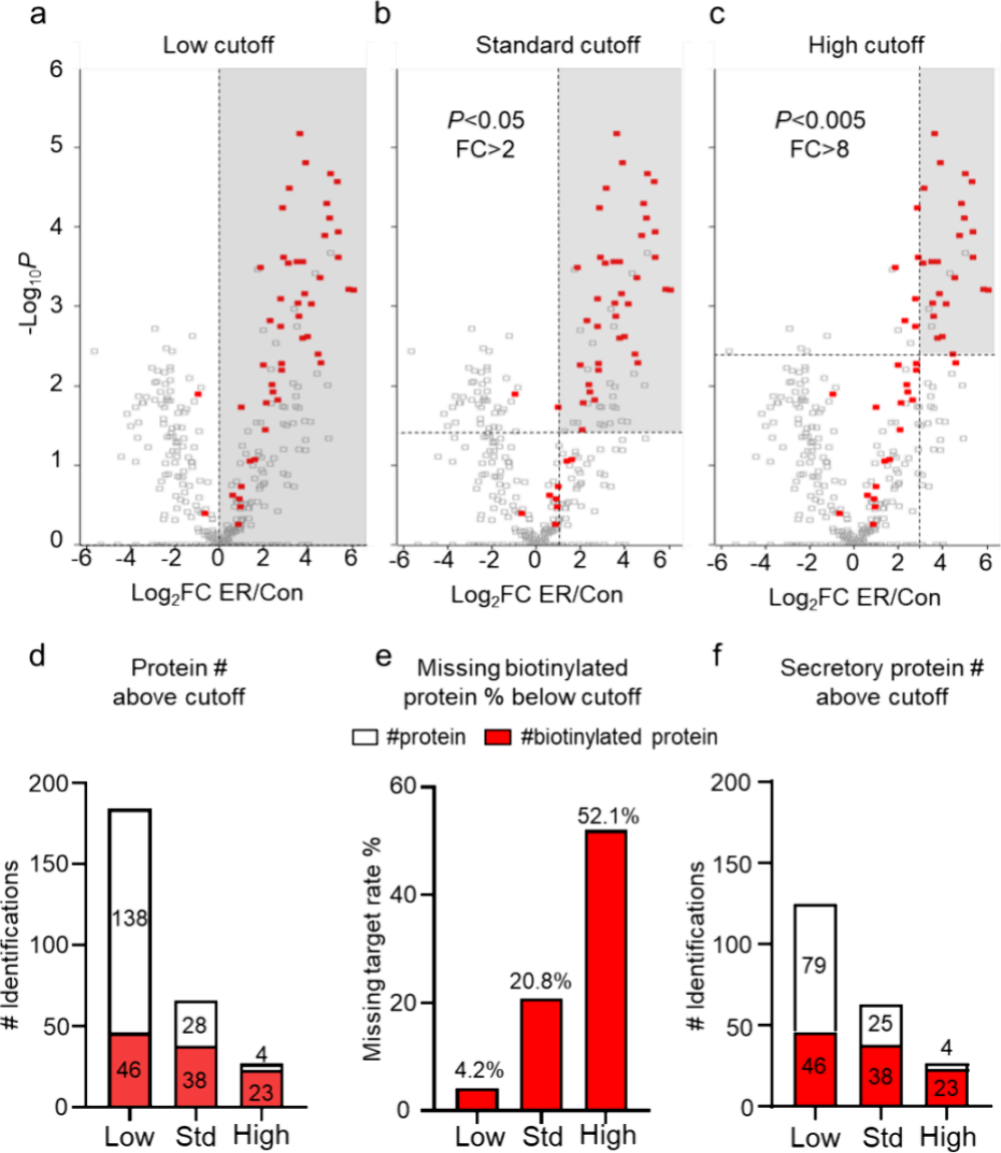
Direct analysis of biotinylated secretome. (a) Volcano plot showing differentially expressed proteins from reanalysis of quantitative LC-MS data from ER-TurboID and control groups with low cutoff (fold change > 0). (b) Volcano plot with standard cutoff (fold change > 2, *p*-value < 0.05). (c) Volcano plot with high cutoff (fold change > 8, *p*-value < 0.005). Red spots indicate biotinylated proteins. (d) Number of proteins identified under each cutoff criterion, with the number of biotinylated proteins highlighted in red. (e) Proportion of biotinylated proteins excluded under each cutoff criterion among all identified biotinylated proteins. (f) Number of secreted proteins identified under each cutoff criterion.

Our results highlighted a significant limitation of conventional statistical approaches, where true positive biotinylated proteins may be incorrectly excluded due to cutoff thresholds. Furthermore, we observed that while all biotinylated proteins were secretory, the nonbiotinylated proteins identified under the traditional statistical approach included some nonsecretory proteins (Figure 3d,f). Collectively, these findings suggest that the direct biotinylation secretome analysis offers a more accurate and reliable method with fewer errors compared with traditional statistical approaches.

### Direct Analysis of Tissue-Specific Secretome

Result data from the in vivo labeling system developed by Wei et al.^13^ for studying the secretome of four different cell types was also included in the data set PXD021602. They designed AAV constructs for tissue-specific TurboID expression and a conditional Cre-dependent flip-excision (FLEx) AAV strategy to establish an in vivo labeling system (Figure 4a). Blood samples were prepared and analyzed by LC-MS, following the same protocols as described above. Our analysis revealed that while approximately 200–400 proteins were identified in the secretome of each cell type, only a small fraction of these were identified as directly biotinylated proteins (Figure 4b). In particular, about 35% of all proteins identified from hepatocytes were biotinylated, indicating a relatively high degree of biotinylation-mediated secretion into the blood via TurboID. In contrast, only 2–7% of proteins identified from myeloid cells, pericytes, and myocytes were biotinylated, suggesting a lower degree of biotinylation and secretion into the bloodstream compared to hepatocyte-derived proteins (Figure 4c). These findings suggest that direct biotinylation secretome analysis can reveal results in the secretome that are not detectable using conventional methods, which do not consider the biotinylation status.

**Figure 4:**
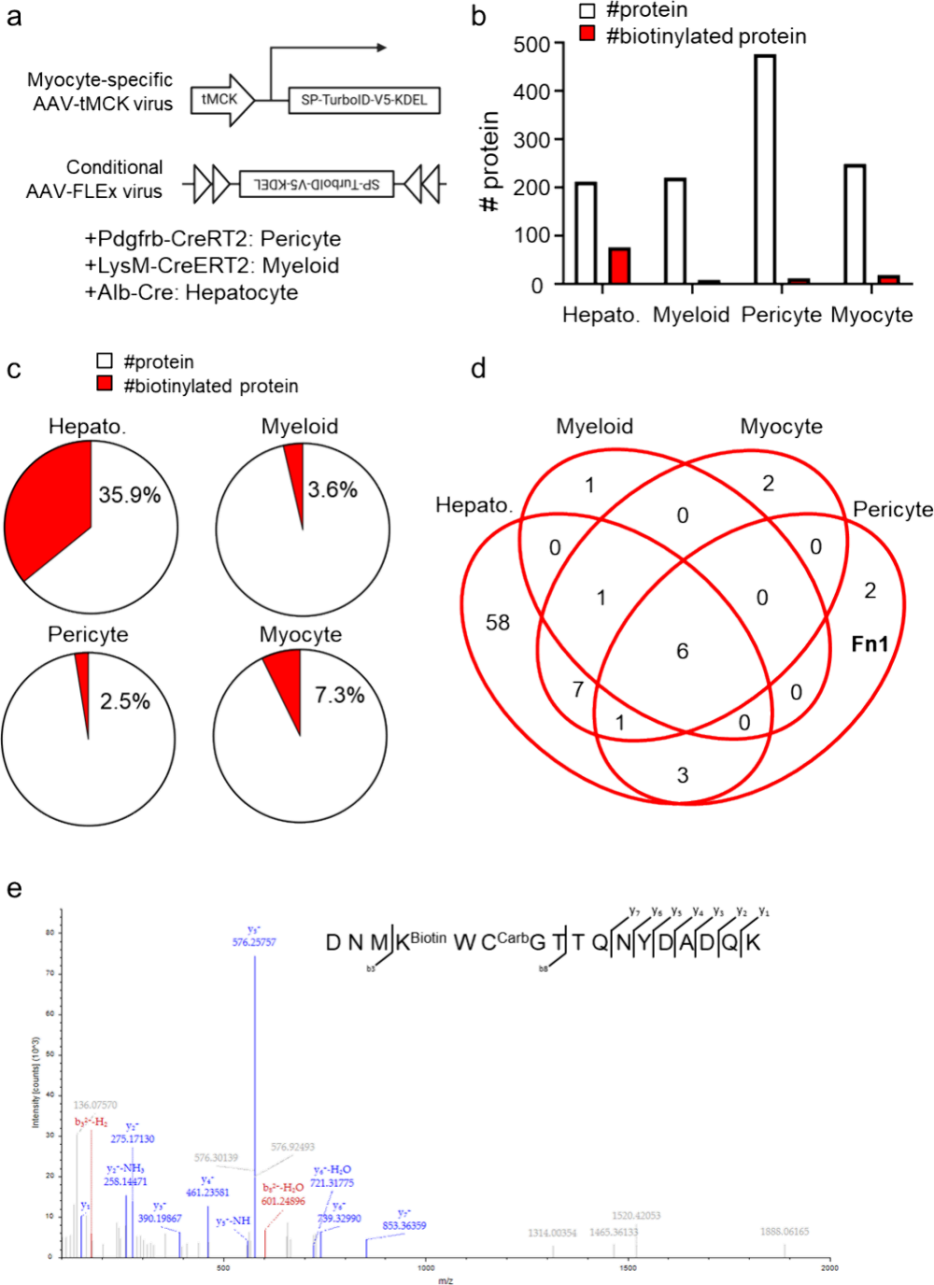
Direct analysis of the tissue-specific secretome. (a) Schematic representation of the tissue-specific TurboID-expressing AAV constructs. These constructs were designed to achieve TurboID expression specifically in target tissues for in vivo secretome labeling. (b) Number of proteins identified through reanalysis for each tissue-specific in vivo secretome labeling system. White bars indicate the total proteins identified, while red bars represent biotinylated proteins. (c) Pie charts showing the proportion of biotinylated proteins among the total identified proteins for each tissue-specific in vivo secretome labeling system. (d) Venn diagram illustrating the overlap of biotinylated proteins identified across different tissue-specific in vivo secretome labeling systems. (e) MS/MS spectrum of the biotinylated peptide derived from the FN1 protein, identified as a pericyte-specific secreted protein through reanalysis of the data.

Through a comparative analysis of biotinylated proteins identified in each cell type, we were able to distinguish shared proteins and cell-type-specific secreted proteins (Figure 4d). While Wei et al.^13^ identified diverse immunoglobulins (KV5A6) as a pericyte-selective secretome marker, our direct biotinylation analysis indicated that although KV5A6 was present in the pericyte-derived secretome, it was not biotinylated, suggesting it may be a false positive. Instead, we identified FN1 as a pericyte-specific secreted protein. FN1 was detected in the secretomes of all cell types; however, biotinylated FN1 was exclusively observed in the pericyte-derived secretome, allowing us to designate FN1 as a selective marker for pericytes (Table S1). Mass spectrometry analysis of biotinylated FN1 peptides provided high-confidence evidence of FN1 biotinylation (Figure 4e). Although FN1 was also identified in Wei et al.’s^13^ analysis, it was not suggested as a pericyte-specific marker using their statistical approach.

These findings highlight the limitations of conventional methods for studying the biotinylated proteome and demonstrate that our proposed direct biotinylation secretome analysis offers a valuable and relatively accurate alternative. As the field of biotinylation-based research continues to expand, this analytical approach should be widely adopted to enable more reliable and robust research outcomes.

## Discussion

In this study, we reanalyzed data derived from a previous study that employed conventional data analysis methods. Using our direct biotinylation analysis approach, we successfully identified biotinylated peptides with higher reliability and confidence compared with the original analyses. This highlights the robustness of our method in enhancing the detection of biotinylated peptides and improving the overall quality of proteomic data interpretation. While we aimed to validate our approach further by applying it to similar studies, our efforts were constrained by a lack of accessible raw data. Many relevant studies had not deposited their data in public repositories such as PRIDE, and even when raw data were available, critical metadata, such as details about sample labeling, were frequently missing. These limitations underscore the necessity for standardized data deposition practices and comprehensive metadata reporting to facilitate reanalysis and reproducibility, ultimately advancing the field of proteomics.

Based on our study, we demonstrated the importance of the identification of biotinylated proteins through the direct detection of biotinylated peptides rather than relying on conventional statistical cutoffs for biotinylated protein identification. Even when biotinylated peptides are enriched during LC-MS-based biotin proteomics workflows, biotinylated peptides typically constitute only about 20% of the total identified peptides. This low biotinylated peptide proportion increases the risk of false positives when using conventional cutoff-based statistical methods. A major contributor to this limitation is the insufficient data quality generated from negative controls, often derived from WT (nonbiotinylated cells), which are commonly used in various studies. While it is important to include controls lacking the labeling enzyme to filter out endogenously biotinylated or nonspecifically bound proteins, a more reliable approach involves designing experiments where both experimental and control groups are subjected to TurboID-mediated biotinylation. This ensures the more accurate identification of biologically significant biotinylated proteins. Comparative analyses, such as Vehicle vs Drug, Cytosol vs ER, or Sedentary vs Running,^16^ provide more robust controls. Similarly, for protein–protein interaction studies, comparisons like organelle targeting versus protein targeting, such as MTS-TurboID versus Rtn4ip1-TurboID,^29^ are recommended. This approach minimizes false positives and enhances the biological relevance of the findings.

Despite the numerous advantages of our direct biotinylation analysis approach, there are several important considerations that must be considered when applying this method. First, some lysine residues may not be sterically accessible for biotinylation. Second, biotinylated peptides may fall outside the optimal size range for detection by LC–MS/MS because lysine biotinylation has been shown to reduce trypsin digestion efficiency,^30^ potentially leading to longer peptides that are less efficiently identified. Third, biotinylation itself can negatively impact peptide ionization and detection.^31^ Taken together, these factors suggest that our data reanalysis may have applied overly stringent criteria, and therefore, more consideration should be given to the potential for false-negative results.

We propose that the proximity labeling field adopt more transparent and standardized data-sharing practices to enhance reproducibility and reliability. Specifically, researchers should deposit raw data with clear and comprehensive annotations in public repositories such as PRIDE. Since biotinylation analysis is essentially a type of PTM analysis, proper experimental execution ensures that the analyses are straightforward and accessible. By providing detailed metadata and ensuring data completeness, these practices would facilitate more accurate reanalyses and broader application of proximity labeling techniques. Moreover, the integration of these standards into routine workflows will greatly enhance their utility in uncovering critical molecular interactions. Standardizing these approaches will ultimately promote transparency and reproducibility, which will benefit the entire scientific community.

## Methods

### Database Search Analysis for LC-MS/MS Data

Downloaded MS/MS raw data were analyzed by Proteome Discoverer (v 2.3.0.523; Thermo Fisher Scientific) for the identification and label-free quantification of proteins and peptides. For the spectrum selector, the signal-to-noise threshold was set to 1.5. For peak filtering in the spectrum, the top 10 peaks were selected within the 100 Da mass window. The MS/MS data were then analyzed using SEQUEST algorithms against a reviewed mouse UniProt database (released in Apr. 2024). The proteolytic enzyme was set to trypsin (full) with two maximum missed cleavage sites. The minimum peptide length was set to 6, and the maximum peptide length was set to 35. Precursor mass tolerance and fragment mass tolerance were set to 10 ppm and 0.02 Da, respectively. For spectrum matching, both weights of b ions and y ions were set to 1. For dynamic modification search, Oxidation (+15.99492 Da) at methionine, acetylation (+42.01057 Da) at lysine and N-terminal, biotin (+226.0776 Da) at lysine, Met-loss (−131.04048 Da) at methionine, and Met-loss+Acetyl (−89.02992 Da) at methionine were set. For the static modification search, carbamidomethyl (+57.0215) was set. For peptide-spectrum match validation, the Percolator algorithm was used to optimize the number of true hits with a target FDR­(strict) of 0.01 and a target FDR­(relaxed) of 0.05. For MS1-based label-free quantification, the Minora Feature Detector algorithm was used. For peptide filtering, the minimum peptide length was set to 6. For protein filtering, the minimum number of peptides per protein was set to 1. In the protein FDR validator node, both target FDR­(strict) and target FDR­(relaxed) were set to 0.01. For label-free quantification in the consensus step, the feature mapper node and precursor ion quantifier were used. For the feature mapper, the maximum RT shift and mass tolerance of chromatographic alignment were set to 10 min and 10 ppm, respectively. RT tolerance, mass tolerance, and minimum signal-to-noise threshold of feature linking and mapping were set to 2 min, 5 ppm, and 5, respectively. For the precursor ion quantifier, unique + razor was used for peptide quantification, and considering protein groups for peptide uniqueness was set. Precursor abundance quantification was based on the intensity. All peptides were used for normalization and protein roll-up. For protein abundance calculations, summed abundances were used.

### Quantitative Analysis of Identified Proteins

The protein table produced by database search analysis was used for normalization and statistical analysis by Perseus 2.0.11.0 software (https:// maxquant.net/perseus/). The abundance intensities were normalized by dividing each protein value (row) by the median of the values of the corresponding protein (row). The normalized abundance intensities were transformed into a Log_2_ scale. After the transformation, additional normalization was performed by subtracting transformed values in each sample (column) from the median transformed value of the corresponding sample (column). The averages of normalized and transformed values for each group were calculated and used to determine the Log_2_ fold changes by subtraction between groups. Student’s two-sample *t*-test was used to calculate *p*-values for comparisons between groups. Biotinylated proteins were selected by filtering proteins based on the modification column.

## Supplementary Material





## Data Availability

Proteomics data have been deposited to the PRIDE server under accession code PXD021602.

## Notes

Information on proteins identified through reanalysis of the mass spectrometry data generated from PXD021602;^13^ summary table is included, showing the number of proteins identified in each category; additional tables provide detailed information on proteins derived from the hepatocyte in vivo subcellular secretome labeling system or the tissue-specific in vivo secretome labeling system, including basic protein information, SignalP 5.0 analysis results, and various mass spectrometry metrics; all terms used in the table are derived from Thermo Proteome Discoverer version 3.2.0.450; #protein unique peptides indicates the total number of distinct peptide sequences unique to the individual protein; #unique peptides indicates the total number of distinct peptide sequences unique to the protein group; #PSMs indicate the total number of peptide-spectrum match; table presents information on peptides identified through reanalysis of the mass spectrometry data generated from PXD021602 ^13^ (XLSX)
Summary table provides the number of peptides identified in each category; additional tables include detailed information on peptides derived from the hepatocyte in vivo subcellular secretome labeling system or the tissue-specific in vivo secretome labeling system, along with various mass spectrometry analysis results; all terms used in the table derived from Thermo Proteome Discoverer version 3.2.0.450 (XLSX)
